# Pulmonary tumours found in a British abattoir survey: primary carcinomas in cattle and secondary neoplasms in cattle, sheep and pigs.

**DOI:** 10.1038/bjc.1968.7

**Published:** 1968-03

**Authors:** L. J. Anderson, A. T. Sandison

## Abstract

**Images:**


### PULMONARY TUMOURS FOUND IN A BRITISH ABATTOIR SURVEY: PRIMARY CARCINOMAS IN CATTLE AND SECONDARY NEOPLASMS IN CATTLE, SHEEP AND PIGS

LINDSAY J. ANDERSON AND A. T. SANDISON

*From the Department of Experimental Veterinary Medicine, University of Glasgow, and the University Pathology Department, Western Infirmary, Glasgow*

Received for publication December 5, 1967

PRIMARY epithelial tumours of the lung in man have been the subject of much interest and extensive investigation in recent years, because of the marked increase in incidence of human lung cancer and the consequent search for carcinogenic agents implicated in its aetiology. Comparative studies in animals might be of value in assessing the importance of exposure to environmental carcinogens. However, little is known of the incidence of primary lung tumours in animals, particularly in the large species, and there is no reliable evidence concerning any recent change in frequency. The aetiology of spontaneous lung cancer in animals has not been investigated and there is little evidence of any variation in incidence in rural and industrial areas. In one series of lung tumours in dogs, no difference was found in city and country dogs (Nielsen and Horava, 1960). There have been very few reports of such tumours in food-producing animals in Britain. Recently, we carried out, in collaboration with the Ministry of Agriculture, Fisheries and Food, a survey of all tumours encountered in 100 abattoirs throughout Great Britain during one year. The specimens collected included 25 primary lung cancers in cattle; none were found in sheep or pigs. This paper, the third of a series of reports based on the survey, describes the primary lung tumours in cattle and lists the secondary lung tumours found in the 3 species.

### MATERIALS AND METHODS

One hundred Meat Inspectors in abattoirs throughout Great Britain agreed to participate in a survey of tumours found in slaughtered animals during the year October 1965 to September 1966. They submitted tissue from all suspected neoplasms found in cattle, sheep and pigs to the University of Glasgow Veterinary Hospital for histological diagnosis. The specimens were fixed in 10% formol-saline in the abattoir and sent in polythene-lined bags, with an accompanying form, giving the owner's name and address, the abattoir address and details of the species, breed, age, sex of the animal and the organs seen to be affected. In the laboratory, the specimens were trimmed, further fixed for 24 hours in formol-saline and processed for paraffin section in the usual way. Sections from all blocks were stained with haemalum and eosin (H. & E.). Special stains used on selected sections included Masson's trichrome, Van Gieson, Gordon and Sweet reticulin stain, phosphotungstic acid haematoxylin (P.T.A.H.), periodic acid Schiff (PAS) and Southgate's mucicarmine methods.

### RESULTS

### *Incidence of lung tumours*

The total numbers of animals slaughtered during the year in the 100 abattoirs participating in the survey included 1·3 million cattle, 4·5 million sheep and 3·7 million pigs. From the records of the Ministry of Agriculture, Fisheries and Food, it was found that these numbers represent approximately one third of all cattle, sheep and pigs slaughtered throughout Great Britain in this period. A total of 713 specimens was submitted for examination from the abattoirs and 548 of these were diagnosed as true neoplasms. In cattle, 25 primary carcinomas of lung were received, giving an incidence of 8·3% of all tumours, occurring at a rate of 19 per million cattle slaughtered. No lung cancers were encountered in sheep and pigs, though 1 specimen of ovine pulmonary adenomatosis was included in the series. The incidence of lung cancer in relation to the numbers of animals surveyed and to the total number of tumours examined is shown in Table I.

TABLE I.—*Incidence of Pulmonary Neoplasms in Slaughtered Cattle, Sheep and Pigs*

| | No. of animals surveyed | No. of tumours examined | No. of primary lung cancers | No. of secondary lung tumours |
|---|---|---|---|---|
| Cattle | 1·3 million | 302 | 25 (8·3% of all tumours) | 55 |
| Sheep | 4·5 million | 107 | 0 (1 Adenomatosis) | 11 |
| Pigs | 3·7 million | 139 | 0 | 14 |

Since the literature contains few reports of bovine lung cancer, the main features of the 25 examples in this series are listed individually in Table II. All of the tumours were from females (the adult cattle population consists almost entirely of cows) and a wide age incidence was found. The youngest animal was only 14 months old but the great majority were adults of more than 5 years of age. Almost all of the carcinomas had metastasised. The most common sites of metastasis were the broncho-mediastinal lymph nodes (18 cases); other nodes in the thorax and abdomen were often involved and sometimes peripheral nodes were also affected. Metastases were frequently noted in the liver (12 cases), pleura (9 cases), kidneys (7 cases), adrenals (4 cases) and peritoneum (3 cases). In one case the spinal canal, eye and ovary were invaded.

There was no evidence of any regional incidence of bovine lung cancer. The farms from which the affected animals originated were in widely scattered rural areas and, although the location was not known accurately in every case, none of our specimens was received from heavily industrialised districts.

The secondary tumours found in lung in the 3 species are listed in Table III. In cattle, secondary deposits were more than twice as common as primary tumours. However, in the cases of fibrosarcoma, the primary site was uncertain and in one instance, only the lung was seen to be involved by the Meat Inspector. Nevertheless, the evidence for such tumours originating in lung was tenuous and they will be included in a later paper describing tumours of soft tissues. In the 3 species, lymphosarcoma was the most frequent secondary tumour of the lung, which, in our experience, is often infiltrated in disseminated lymphosarcoma.

TABLE II.—*Details of 25 Cases of Bovine Bronchial Carcinoma*

| No. | Breed | Age | Histological type | Sites of metastases |
|---|---|---|---|---|
| 1 (90) | Friesian | 6 yr | Adenoca. of columnar-cell acinar type | No metastases noted |
| 2 (249) | S. Devon | 8 yr | Adenoca. of well-differentiated columnar-cell acinar type | Liver |
| 3 (266) | Friesian | Adult | Adenoca. with columnar-cell acinar structure and some anaplastic areas | Broncho-mediastinal lymph nodes |
| 4 (76) | Welsh Black | > 10 yr | Papillary adenoca., well differentiated | Widespread lymph node involvement; liver; peritoneum |
| 5 (407) | Friesian | 6 yr | Papillary acidophilic adenoca. | Pleura |
| 6 (689) | Ayrshire | > 10 yr | Papillary acidophilic adenoca. | Pleura |
| 7 (146) | Friesian | > 12 yr | Squamous ca. of epidermoid type | Many lymph nodes; liver; kidneys |
| 8 (533) | Shorthorn | > 10 yr | Squamous carcinoma, heavily keratinising | Pleura |
| 9 (459) | Devon | 5 yr | Oat-cell carcinoma with some acinar differentiation in places | Many lymph nodes |
| 10 (144) | Shorthorn | 2½ yr | Spindle-cell ca. with stromal reaction | Broncho-mediastinal lymph nodes |
| 11 (129) | Ayrshire | 6 yr | Polygonal celled anaplastic ca. with occasional mucicarmino-philic cells | Many lymph nodes; pleura |
| 12 (190) | Shorthorn | > 10 yr | Anaplastic polygonal cell ca. with spindle cell areas. Calcispherites present | Thoracic and prescapular lymph nodes; pleura |
| 13 (123) | Friesian | > 10 yr | Anaplastic, pleomorphic carcinoma | Pleura |
| 14 (131) | Shorthorn | > 10 yr | Anaplastic, pleomorphic carcinoma | Thoracic lymph nodes |
| 15 (173) | — | 8 yr | Anaplastic, pleomorphic carcinoma | Bronchomediastinal nodes; liver; lumbar mass |
| 16 (193) | — | 14 mth | Anaplastic, pleomorphic carcinoma | Pleura; liver; adrenals; ovary; spinal canal |
| 17 (291) | Ayrshire | 10 yr | Anaplastic, pleomorphic carcinoma | Bronchomediastinal, hepatic, lumbar and mes-enteric nodes; liver; kidneys; adrenals |
| 18 (98) | — | 6 yr | Ca. with both squamous and adenocarcinomatous differentiation | Many lymph nodes; liver; kidneys; adrenals |
| 19 (206) | Friesian | 8 yr | Ca. with both squamous and adenocarcinomatous differentiation | Bronchial lymph nodes; pleura; liver; kidneys |
| 20 (366) | — | Adult | Ca. with acinar and squamous elements | Widespread lymph node involvement; kidneys |
| 21 (440) | — | 6 yr | Anaplastic ca. with poorly differentiated squamous and acinar areas | Bronchomediastinal lymph nodes |
| 22 (118) | Shorthorn | 5 yr | Poorly differentiated adenoca. with undifferentiated and spindle cell areas | Many lymph nodes; liver; kidneys |
| 23 (210) | — | Adult | Poorly differentiated adeno-carcinoma with anaplastic spindle-cell areas | Bronchomediastinal and hepatic lymph nodes; liver |
| 24 (601) | Friesian | 12 yr | Ca. with distinctive oat-cell, spindle-cell and acinar areas | Thoracic, prescapular and mesenteric nodes; liver; kidneys; adrenals; eye |
| 25 (691) | — | 6 yr | Ca. with oat-cell, spindle-cell and acinar areas | Bronchomediastinal nodes; peritoneum |

TABLE III.—*Secondary Tumours in Lung in Cattle, Sheep and Pigs*

| Cattle Total = 55 | Sheep Total = 11 | Pigs Total = 14 |
| --- | --- | --- |
| 20 Lymphosarcoma | 7 Lymphosarcoma | 13 Lymphosarcoma |
| 8 Fibrosarcoma | 1 Fibrosarcoma | 1 Ovarian ca. |
| 6 Squamous ca. from eye | 1 Haemangio-endothelioma | |
| 5 Cholangioca. | 1 Cholangioca. | |
| 3 Liver-cell ca. | 1 Renal ca. | |
| 2 Granulosa-cell ca. | | |
| 2 Renal ca. | | |
| 2 Reticulosarcoma | | |
| 1 Thyroid ca. | | |
| 1 Nephroblastoma | | |
| 1 Chondrosarcoma | | |
| 1 Cervical ca. | | |
| 1 Ovarian ca. | | |
| 1 Neurofibrosarcoma | | |
| 1 Reticulosis, possibly of lipid-storage type | | |

*Histological appearances*

Considerable variation was seen in the histological structure of the bovine lung cancers. The majority were essentially either adeno-carcinomas or squamous carcinomas showing a greater or lesser degree of differentiation, with some tumours classifiable only as anaplastic or pleomorphic forms when no evidence of morphological differentiation was found. The series included well-differentiated adenocarcinomas of acinar, columnar-cell type (cases 1–3), papillary adenocarcinomas (cases 4–6), one epidermoid squamous carcinoma (case 7), 1 heavily keratinising squamous carcinoma (case 8), oat-cell and spindle-cell forms (cases 9 and 10), polygonal-cell solid carcinomas (cases 11 and 12) and several anaplastic carcinomas (cases 13–17). A number of the tumours showed a transition between 2 or more histological types: in 4, there were both mucin-secreting adenocarcinomatous and squamous elements in the same tumour (cases 18–21) and in 4 there were oat-cell and spindle cell areas and foci of adenocarcinomatous differentiation (cases 21–25). The principal morphological characteristics of each tumour in the series are indicated in Table II.

The well-differentiated adenocarcinomas were composed of non-ciliated columnar epithelium with acidophilic cytoplasm arranged in tubules and acini (Fig. 1). There was no scirrhous reaction. The papillary carcinomas also consisted of acidophilic cells (unlike renal papillary tumours, which tend to be basophilic) and these were arranged in a regular, clearly-defined branching pattern (whereas renal papillary cancers show solid masses with a more simple and less regular branching pattern) (Fig. 2). The epithelium varied from cuboidal to columnar in the individual examples, but tended to be uniform in a given tumour. Some degree of mucin secretion was a feature of all of the adenocarcinomas.

The epidermoid squamous carcinoma was formed by solid branching trabeculae and islands of squamous epithelium which were sharply delineated and contained central foci of necrosis, giving a striking pattern (Fig. 3). Keratin deposition was not present but some cells close to the central necrotic zones showed intra-cytoplasmic keratinisation. The necrotic debris had the strongly acidophilic appearance characteristic of necrosis in a squamous tumour. The keratinising squamous carcinoma was unlike the epidermoid tumour in having no regular arrangement. Ill-defined sheets of squamous cells streamed through the lung parenchyma with areas of heavy keratin deposition. In places, keratin breakdown had occurred and an associated inflammatory reaction with giant cells was present.

The carcinomas of purely or partially oat-cell type were solid tumours, composed of dense masses of basophilic short blunt oval cells with large hyperchromatic nuclei and a high mitotic rate (Fig. 4). Occasional cells contained PAS-positive granules or small globules. Lymphatic invasion was a marked feature. In 1 case, classified as a spindle-cell cancer, the cells were particularly slender and tapered in shape and this tumour showed an unusual degree of stromal reaction. Small groups of tumour cells were separated by intervening bands of fibrous tissue, producing a regular lattice arrangement (Fig. 5). Within lymphatics, however, there were dense masses of cells with the more typical oat-cell appearance.

The 2 polygonal-cell carcinomas were considered to be essentially anaplastic tumours, showing no architectural pattern and little cellular differentiation. They were solid carcinomas, formed by masses of polygonal cells with faintly acidophilic cytoplasm, large vesicular nuclei and prominent nucleoli (Fig. 6). Reticulin was scanty. In 1, the cells were PAS and mucicarmine-negative and small calcipherites were scattered throughout the tumour. In the other, occasional mucin-containing cells were demonstrated. Direct invasion of alveoli and permeation of lymphatic vessels were apparent. In places, the cells became somewhat spindle shaped. The 5 specimens of pleomorphic, anaplastic carcinoma were alike in being composed of dense masses of conjoined cells with large hyperchromatic nuclei and basophilic cytoplasm. There was much variation in the size and shape of the cells within the tumours, including spindle, oval and polygonal forms. Multinucleated giant cells were seen in one case. The mitotic rate was high. In some features, such tumours resembled lymphoid neoplasms in H. & E. sections: however, a well-developed reticulin network was demonstrable, surrounding packets of tumour cells in an epithelial pattern and occasional cells contained PAS-positive granules or globules. The degree of pleomorphism was greater than is usual in bovine lymphoid neoplasms. A further characteristic common to all of the oat-cell and anaplastic carcinomas was the presence of discrete areas of necrosis in the centre of groups of tumour cells. The appearance of central zones of necrotic debris bounded by a band of viable carcinoma advancing directly into the adjacent lung parenchyma was a typical finding in this group. The necrosis pattern also helped to differentiate the anaplastic basophilic cancers from lymphoid tumours.

The carcinomas in which more than 1 morphological variant occurred were of particular interest. In 4 cases, tumours of adenoacanthoma type were composed of groups of cuboidal or columnar cells forming acini and showing mucin secretion, in close apposition to solid whorls of squamous cells which sometimes showed intracellular keratinisation (Fig. 7 and 8). In 1 example, the mucin-secreting cells were of signet-ring form. In 4 others, areas of adenocarcinomatous differentiation were found in tumours of mainly oat-cell or undifferentiated type.

The sections of lung tissue were also examined for evidence of any associated disease process. In 11 cases, no lung unaffected by tumour was present in the available material. In 3 cases (144, 190 and 366) the parenchyma adjacent to the cancer was normal. In 5 (98, 118, 131, 146 and 601) a marked degree of pulmonary oedema was present, with extensive fibrin deposition in 1 case. One (249) showed pulmonary oedema and early bronchopneumonia and in another (291) a foreign-body reaction to an aspirated oily material was seen. Scar formation was present in 4 (90, 193, 266 and 553) associated with unresolved bronchopneumonia and in one of these (193) there was squamous metaplasia in a bronchus and ossification in the scar tissue.

The single specimen of ovine pulmonary adenomatosis (" Jaagsiekte ") was from a 1 year old female. Although there remains doubt as to whether this is a truly neoplastic disease, we include the case for completeness and for comparison with the microscopic appearances of lung cancer. Histologically, the well-known changes characteristic of the condition were present, producing a uniform lining of the alveoli by a regular single layer of acidophilic columnar cells. Sometimes these cells formed papillary ingrowths into the alveoli. Mitotic figures were uncommon. Many alveoli contained macrophages and there was an interstitial inflammatory reaction in which mononuclear cells predominated. The bronchial lymph nodes were not noted by the Meat Inspector to be affected and were not received for examination.

### DISCUSSION

In this study of tumours submitted from abattoirs the diagnosis of primary carcinoma of lung presented some difficulty, as we had no indication of the relationship of the tumours to the bronchial tree, nor, in disseminated cases, which affected organ contained the major tumour mass. Since certain secondary malignancies in the lung may simulate primary cancers and occult carcinoma of lung may produce widespread and massive metastases, care was taken to eliminate other possible primary tumours before making the diagnosis. In general, we have followed the accepted diagnostic criteria of human pathology, based on histological appearances and distribution of metastases (Evans, 1966; Liebow, 1952; Willis, 1960). We are satisfied that the origins of the secondary tumours found in lung were readily recognisable histologically. However, in the absence

---

### EXPLANATION OF PLATES

Fig. 1.—Well-differentiated adenocarcinoma of acinar pattern, composed of eosinophilic columnar cells. Anaplastic carcinoma is present in alveoli in the top of the field. H. & E. ×100.

Fig. 2.—Adenocarcinoma of papillary structure, showing a regular branching pattern formed by eosinophilic low columnar cells. H. & E. ×110.

Fig. 3.—Epidermoid squamous carcinoma, showing broad trabeculae of squamous cells with central zones of necrosis. H. & E. ×110.

Fig. 4.—Oat-cell carcinoma, showing dense masses of basophilic oval cells invading alveoli. H. & E. ×100.

Fig. 5.—Spindle-cell carcinoma. In this tumour, the cancer cells are unusually slender and tapered in shape and a marked stromal reaction produces a distinctive lattice pattern. H. & E. ×110.

Fig. 6.—Polygonal-cell carcinoma. Essentially an anaplastic carcinoma, here showing masses of conjoined eosinophilic polygonal cells invading alveoli. H. & E. ×250.

Fig. 7.—Carcinoma with closely related squamous and adenocarcinomatous elements. The tumour is composed of packets of squamous cells with focal acinar differentiation, as shown here, and mucin secretion. H. & E. ×110.

Fig. 8.—Carcinoma in an area of unresolved pneumonia. This tumour contains both squamous and adenocarcinomatous elements with irregular formation of tubules and acini. Occasional cells are mucicarminophilic while many, particularly in the solid groups, are of squamous type. Masson ×110.

1

2

3

4

5

6

Anderson and Sandison.

7

8

Anderson and Sandison.

of detailed necropsy examinations, the series of tumours described in this paper can be classified as carcinoma of lung only with a high degree of probability and not with absolute certainty. From the published records of the incidence and types of bovine neoplasia, the diagnosis in two of our cases may be questioned. The carcinoma from a 14 month-old heifer was surprising at this age; the ovary was affected and primary ovarian tumours are known to occur in young cattle. However, the lesion in the ovary was clearly a secondary deposit and had no histological resemblance to recognised ovarian tumours. The keratinising squamous tumour was reminiscent of the well-known eye cancer of cattle, but a tumour of the eye would almost certainly reach considerable size before producing widespread pulmonary metastases and in this case, no eye lesion was noted. Attention has been drawn to the possibility of confusing secondary carcinoma of the uterus in lung with primary lung cancer in cattle (Monlux, Anderson, Davis and Monlux, 1956). To avoid missing any specimens of uterine carcinoma, at the start of the survey we specially requested the Meat Inspectors to examine the uteri with particular care. This study has not revealed any carcinomas of uterus and our experience at the University of Glasgow Veterinary Hospital also suggests that it is a very rare tumour in Britain. The adenocarcinomas in the present group differed from the published description of uterine cancer (Monlux, Anderson, Davis and Monlux, 1956) most distinctly in the absence of scirrhous reaction and the presence of anaplastic change in most instances.

Many pathologists (Clegg, 1958; Evans, 1966; Liebow, 1952) agree that in recent years there has been a real and considerable increase in the incidence of bronchial carcinoma in man although occasional authorities have some reservations (Willis, 1960). In most hospital necropsy series, carcinoma of bronchus is one of the most common malignant tumours. There is general agreement that carcinoma of the lung is rare in cattle, sheep and pigs and our observation that it is among the more common epithelial tumours of cattle is at variance with some reports. The literature contains few well-authenticated descriptions of bovine lung cancer and there is little available information concerning the incidence and histological appearances of the tumour in this species. In a review, Krahnert (1954) notes that estimates of the relative incidence of bovine lung cancer vary from 2·8–12·5%. In an abattoir survey in the U.S.A., 186 bovine tumours from approximately 1·4 million slaughtered cattle included only 3 primary carcinomas of lung (Monlux, Anderson and Davis, 1956). Also from the U.S.A., a study of 1000 tumours from abattoirs yielded 21 adenocarcinomas of lung from cattle, as well as 2 from sheep and a small number of mesenchymal tumours (Brandly and Migaki, 1963). A series of 202 bovine tumours from Kansas included 1 lung cancer (Sastry and Tweihaus, 1964). An early study of tumours in the Glasgow abattoir revealed 8 (2·6%) bovine lung cancers (Trotter, 1911). In other series of 293, 90, 108 and 144 bovine tumours, no primary carcinomas of lung were found (Cotchin, 1960; Davis, Leeper and Shelton, 1933; Jackson, 1936; Nobel and Neumann, 1960).

Reports of lung cancer in sheep are scarce; Krahnert (1954) cites 2 references and 2 examples have been recorded from the U.S.A. (Brandly and Migaki, 1963) and 1 from South Africa (Jackson, 1936). The absence of any in our series of 107 ovine tumours further illustrates the rarity of this tumour in sheep. We have not found any published description of lung cancer in pigs and none occurred in our series. Carcinoma of the lung is well-known in dogs and cats (Brodey and Craig, 1965; Ferri and Tausk, 1955; Jenny, 1945; Sedlmeier and Dahme, 1958; Stünzi, 1965; Thije and Ressang, 1956), and estimates of the relative incidence vary from 1·3–7·0% in dogs and 1·3–14% in cats. The tumour is also recognised in horses (Jenny, 1945; Krahnert, 1955; Matthias and Schützler, 1941; Schlegel, 1933; Sjolte, 1944). Lung cancer has been reported in a variety of wild animals and in several species of birds (Snyder and Ratcliffe, 1966). Other rare forms of pulmonary neoplasia have been noted in man (Evans, 1966; Liebow, 1952; Willis, 1960) and in domestic animals. In the latter, these include hamartomas, adenomas, chondromas, leiomyomas, haemangiomas, fibromas and their corresponding sarcomas (Brandly and Migaki, 1963; Nieberle and Cohrs, 1949; Schlegel, 1933). In our series, several mesenchymal tumours affected the lung but because the primary site was uncertain, we shall describe these in a later paper on soft tissue tumours. We did not find hamartomas or adenomas of the lung in the survey.

There is no conclusive evidence of any increase in the incidence of lung cancer in animals in recent years, corresponding to that noted in man. Although an increase in dogs has been claimed (Grollet, 1965; Lombard, 1964; Thije and Ressang, 1956) other writers consider that there is no definite indication of any rising incidence in domestic animals (Brodey and Craig, 1965; Jenny, 1945; Krahnert, 1954). Stünzi (1965) described bronchial carcinomas in dogs and cats in Zürich: during the past decade increasing numbers have been diagnosed but this is attributed to a general rise in the age of the animals at autopsy and not to any true change in frequency of the tumour. Our observed frequency in British cattle is somewhat greater than might be expected from earlier reports. However, the incidence has not previously been determined in this country and there are no suitable figures for comparison.

It is generally accepted that lung cancer affects men very much more often than women. Estimates of the sex ratio vary from 5 : 1 to 8 : 1 (Clegg, 1958; Evans, 1966; Liebow, 1952; Willis, 1960) although *adenocarcinomas* of bronchus are commoner in women. The maximum incidence is in the fifth and sixth decades of life. The age and sex incidence of all neoplasms in food-producing animals is modified by the age at slaughter and the fact that most adult cattle are female. Thus we have no information concerning the susceptibility of male cattle to lung cancer and cannot compare the sex incidence with that in man. Other writers have found no evidence of any sex difference in dogs and cats. Most reported lung cancers have been from mature and old animals and the rarity of the tumour in sheep and pigs may be due in part to the immaturity of the majority of these animals when slaughtered.

Willis (1960) rightly emphasises that there is only 1 biological entity—carcinoma of lung—and states that tumours may show great pleomorphism of histological appearance. Most authorities recognise anaplastic, squamous and adenocarcinomatous variants of human lung cancer (Liebow, 1952; Willis, 1960), while some recognise the oat-cell cancer as a differentiated type (Clegg, 1958; Evans, 1966). It is generally accepted that mixed forms occur. The microscopic appearances of spontaneous carcinoma of the lung in animals are of interest in view of the suggested association in man between carcinomas of squamous and oat-cell types and exposure to carcinogenic agents. There is general agreement in the veterinary literature that most animal cases are columnar-cell adenocarcinomas and that squamous and oat-cell cancers are uncommon. This pattern differs from that characteristic of human lung cancer, where adenocarcinomas, although commoner in women, form only a small proportion of the total. Our experience of bovine lung cancer suggests that while adenocarcinomas are fairly frequent, oat-cell, squamous and anaplastic carcinomas are equally numerous. More than 1 histological structure was often present and a rigid classification seems inapplicable. Krahnert (1955) observed that most animal lung cancers are adenocarcinomas, though squamous, undifferentiated and mixed types occur occasionally in several domestic species. Most of Trotter's (1911) cases were termed round-cell carcinomas, possibly corresponding to some of the anaplastic forms in our series. A scirrhous adenocarcinoma from a cow has been described (Lund, 1924) and " mixed-cell " bovine lung cancers have also been noted (Monlux, Anderson and Davis, 1956). Monlux, Anderson, Davis and Monlux (1956) reported 5 carcinomas from cattle, 4 of which were undifferentiated adenocarcinomas and 1 which contained columnar-cell and undifferentiated areas. Brandly and Migaki's (1963) cases were classified as adenocarcinomas. In 7 bovine lung cancers, Sjolte (1944) noted solid adenocarcinomatous and squamous forms as well as tumours of " adenocarcino-sarcoma " type. In dogs and cats, cancers with mixed adenocarcinomatous and squamous elements as well as epidermoid and anaplastic forms have been described in addition to the more common columnar-cell carcinomas, which may show a papillary structure. Oat-cell lung cancers have seldom been recognised in animals. The bronchiolar or alveolar-cell carcinoma did not occur in our series, although we have seen this type in the dog and cat.

In man, the majority of tumours are central, whereas many animal lung cancers arise peripherally (Krahnert, 1954). In our study, we have no detailed information as to the location of the tumours within the lungs and therefore no indication of any relationship between the anatomical distribution and histological appearance. We feel, however, that the frequent occurrence of tumours containing two or more morphological variants suggests that no distinct subdivision is possible; Moulton (1961) reaches a similar conclusion.

The distribution of metastases in our bovine series approximated to that found in man, with broncho-mediastinal lymph nodes usually involved and frequent spread to other thoracic, abdominal and peripheral nodes. The organs most commonly implicated were the liver, kidneys and adrenals. A similar pattern has previously been noted in animal lung cancer (Krahnert, 1955) except that we found the adrenals to be more often affected. As the brain and bones are not examined closely at meat inspection, we do not know whether these were sites of metastases. There was no indication of any difference in metastatic behaviour between squamous, anaplastic and adenocarcinomatous tumours. In the dog, squamous and anaplastic lung cancers have been observed to metastasise more readily than adenocarcinomas (Brodey and Craig, 1965) and in man it has been stated that (although there are many exceptions) squamous tumours tend to spread to the local nodes and distant metastases in anaplastic tumours by the lymphatics and adenocarcinomas by the blood stream (Liebow, 1952).

We found evidence of unresolved bronchopneumonia and scar formation in several of the lungs and there was often a marked degree of pulmonary oedema in the region of the carcinomas. Suggestions have been made that cancer may supervene following metaplastic changes in the lung due to prolonged inflammation (Sedlmeier and Dahme, 1958) and that a local reduction in oxygenation resulting from chronic reactions predisposes to neoplasia (Sudaric, 1963). We consider that there is no good evidence in favour of such hypotheses. In the present series, there was no sign of inflammatory changes in several cases and when present these seem more likely to be a consequence of the presence of the tumour. Further, the high incidence of inflammatory conditions in the bovine lung suggests that their occurrence in association with carcinoma may be fortuitous. Neither is there any evidence in human pathology that influenza, tuberculosis or bronchiectasis predispose to lung cancer.

The aetiology of spontaneous bovine (and other animal) lung cancer is obscure. There did not appear to be any local clustering of our cases which might suggest a causal environmental factor and there is no reason to suppose that cattle are greatly exposed to any of the recognised carcinogens under normal conditions. However, we were particularly interested to find that the histological types of lung cancer associated with carcinogenic agents in man do occur spontaneously in cattle. In experimental studies, the agents shown to be carcinogenic for the lung include ionising radiations, influenza virus in mice, atmospheric pollutants such as nickel carbonyl, asbestos and benzopyrene, and many chemicals of the aromatic amine groups.

The fact that lymphosarcoma is the outstanding malignancy in all domestic animals is reflected in its predominance among the secondary malignancies in animal lungs. Differences in the frequency of the epithelial tumours are also illustrated by comparing their pulmonary metastases in animals and man.

### SUMMARY

The histological features of 25 primary carcinomas of the lung in cattle are described. The carcinomas were examined in the course of a survey of all tumours found in cattle, sheep and pigs in 100 abattoirs throughout Great Britain during one year. No primary lung cancers were encountered in sheep or pigs, though the series included one example of ovine pulmonary adenomatosis. The total numbers of slaughtered animals surveyed were 1·3 million cattle, 4·5 million sheep and 3·7 million pigs and 302 bovine, 107 ovine and 139 porcine tumours were received from the abattoirs. In cattle, the 25 lung cancers represented 8·3% of all neoplasms, occurring at a rate of 19 per million cattle slaughtered. The group of lung cancers included well-differentiated adenocarcinomas of acinar and papillary structure, squamous and oat-cell forms and several anaplastic carcinomas of polygonal-cell and pleomorphic types. In several specimens, there were transitions between 2 or more histological types within a single tumour. We have no evidence of any association between the anatomical distribution of the tumours within the lungs and their histological characteristics. In all but 1 case, metastases were noted. The usual sites of metastasis were the broncho-mediastinal lymph nodes. Frequently other nodes in the thorax and abdomen were involved, as were the pleura, liver, kidneys and adrenals. The lung cancers were all from females, the majority over 5 years of age. The affected cattle originated from widely scattered rural areas, with no local clustering of cases.

The secondary lung tumours in cattle, sheep and pigs are listed. In cattle, secondary deposits in the lung were more than twice as common as primary carcinomas and in the 3 species, lymphosarcoma was the most frequent secondary malignancy affecting the lungs.

The authors thank the Ministry of Agriculture, Fisheries and Food for their co-operation in organising the survey and providing data concerning numbers of animals slaughtered in the participating abattoirs.

We are indebted also to the Meat Inspectors who submitted the specimens for histopathological examination.

Our thanks are due to Professor W. F. H. Jarrett, Department of Experimental Veterinary Medicine, University of Glasgow, for planning the survey and providing the laboratory facilities.

Mr. N. L. Russell carried out the technical work and Mr. A. Finney took the photographs.

### REFERENCES

BRANDLY, P. J. AND MIGAKI, G.—(1963) *Ann. N.Y. Acad. Sci.*, **108**, 872.

BRODEY, R. S. AND CRAIG, P. H.—(1965) *J. Am. vet. med. Ass.*, **147**, 1628.

CLEGG, J. W.—(1958) 'Trachea, Bronchus and Pleura in Cancer', edited by R. W. Raven. London (Butterworths), Vol. 2, p. 563.

COTCHIN, E.—(1960) *Vet. Rec.*, **72**, 816.

DAVIS, C. L., LEEPER, R. B. AND SHELTON, J. E.—(1933) *J. Am. vet. med. Ass.*, **83**, 229.

EVANS, R. W.—(1966) 'Histological Appearances of Tumours', 2nd edition. Edinburgh (Livingstone).

FERRI, A. G. AND TAUSK, E.—(1955) *J. comp. Path. Ther.*, **65**, 159.

GROLLET, L.—(1965) *Rev. Path. gen. comp.*, **65**, 405.

JACKSON, C.—(1936) *Onderstepoort J. vet. Sci. Anim. Ind.*, **6**, 1.

JENNY, J.—(1945) *Inaug. Diss., Zürich.*

KRAHNERT, R.—(1954) *Dt. tierärztl. Wschr.*, **61**, 449.—(1955) *Mh. Tierheilk.*, **7**, 155.

LIEBOW, A. A.—(1952) ' Tumors of the lower respiratory tract '. In 'Armed Forces Institute of Pathology ', Washington.

LOMBARD, G.—(1964) *Écon. Méd. anim.*, **5**, 303.

LUND, L.—(1924) *Berl. tierärztl. Wschr.*, **40**, 234.

MATTHIAS, D. AND SCHÜTZLER, G.—(1941) *Virchows Arch. path. Anat. Physiol.*, **308**, 243.

MONLUX, A. W., ANDERSON, W. A. AND DAVIS, C. L.—(1956) *Am. J. vet. Res.*, **17**, 646.

MONLUX, A. W., ANDERSON, W. A., DAVIS, C. L. AND MONLUX, W. S.—(1956) *Am. J. vet. Res.*, **17**, 45.

MOULTON, J. E.—(1961) 'Tumors in Domestic Animals '. Berkeley and Los Angeles (University of California Press), p. 116.

NIEBERLE, K. AND COHRS, P.—(1949) ' Lehrbuch der Speziellen Pathologischen Anatomie der Haustiere '. Jena (Gustav Fischer), p. 274.

NIELSEN, S. W. AND HORAVA, A.—(1960) *Am. J. vet. Res.*, **21**, 813.

NOBEL, T. A. AND NEUMANN, F.—(1960) *Refuah vet.*, **17**, 39.

SASTRY, G. A. AND TWEIHAUS, M. J.—(1964) *Indian vet. J.*, **41**, 454.

SCHLEGEL, M.—(1933) *Berl. tierärztl. Wschr.*, **49**, 211.

SEDLMEIER, H. AND DAHME, E.—(1958) *Berl. Münch. tierärztl. Wschr.*, **71**, 416.

SJOLTE, I. P.—(1944) *Virchows Arch. Path. Anat. Physiol.*, **312**, 35.

SNYDER, R. L. AND RATCLIFFE, H. L.—(1966) *Cancer Res.*, **26**, 514.

STÜNZI, H.—(1965) *Schweiz. med. Wschr.*, **95**, 1744.

SUDARIC, F.—(1963) *Veterinaria, Saraj.*, **12**, 197.

THIJE, J. H. TEN AND RESSANG, A.—(1956) *Dt. tierärztl. Wschr.*, **63**, 17.

TROTTER, A. M.—(1911) *J. comp. Path. Ther.*, **24**, 1.

WILLIS, R. A.—(1960) ' Pathology of Tumours '. 3rd edition. London (Butterworths).


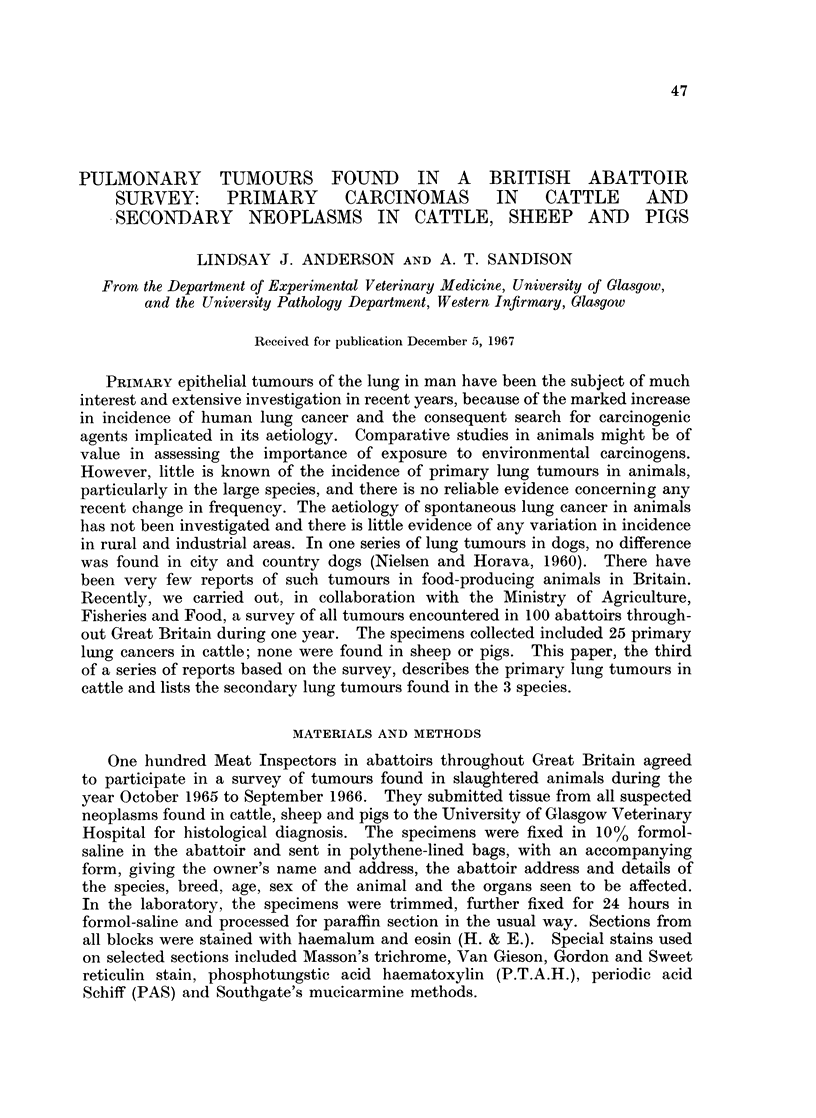

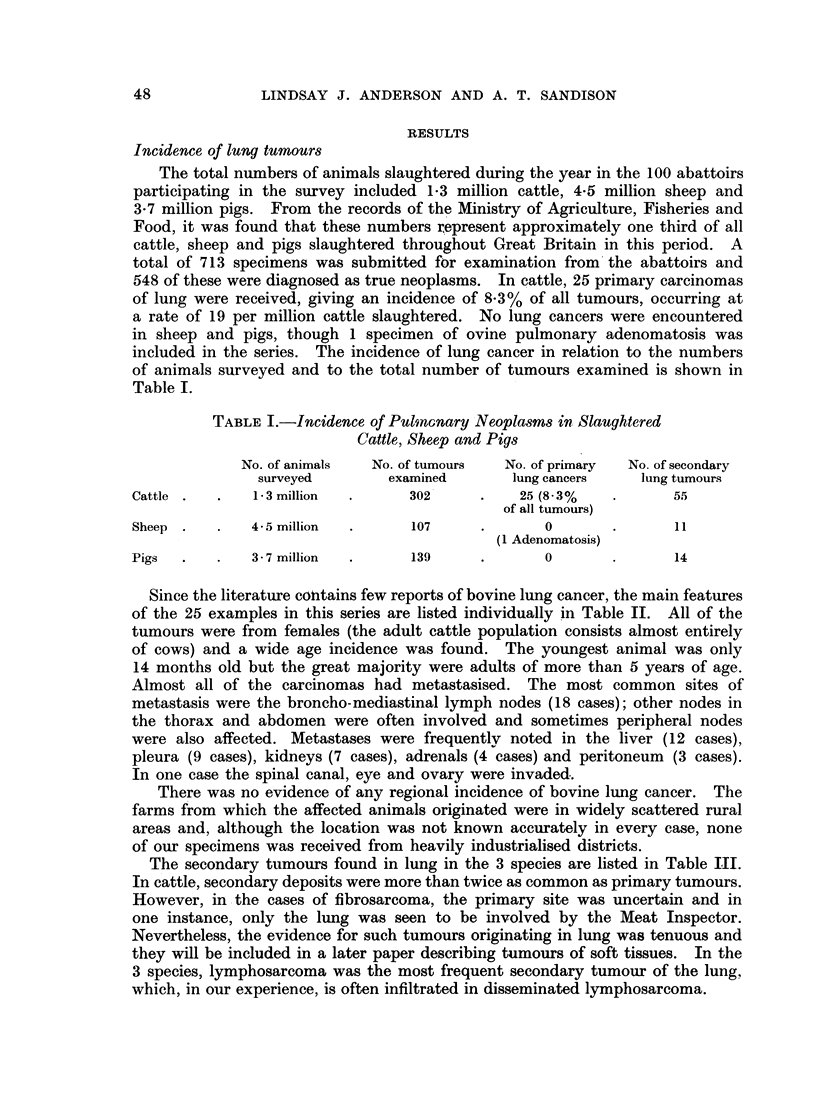

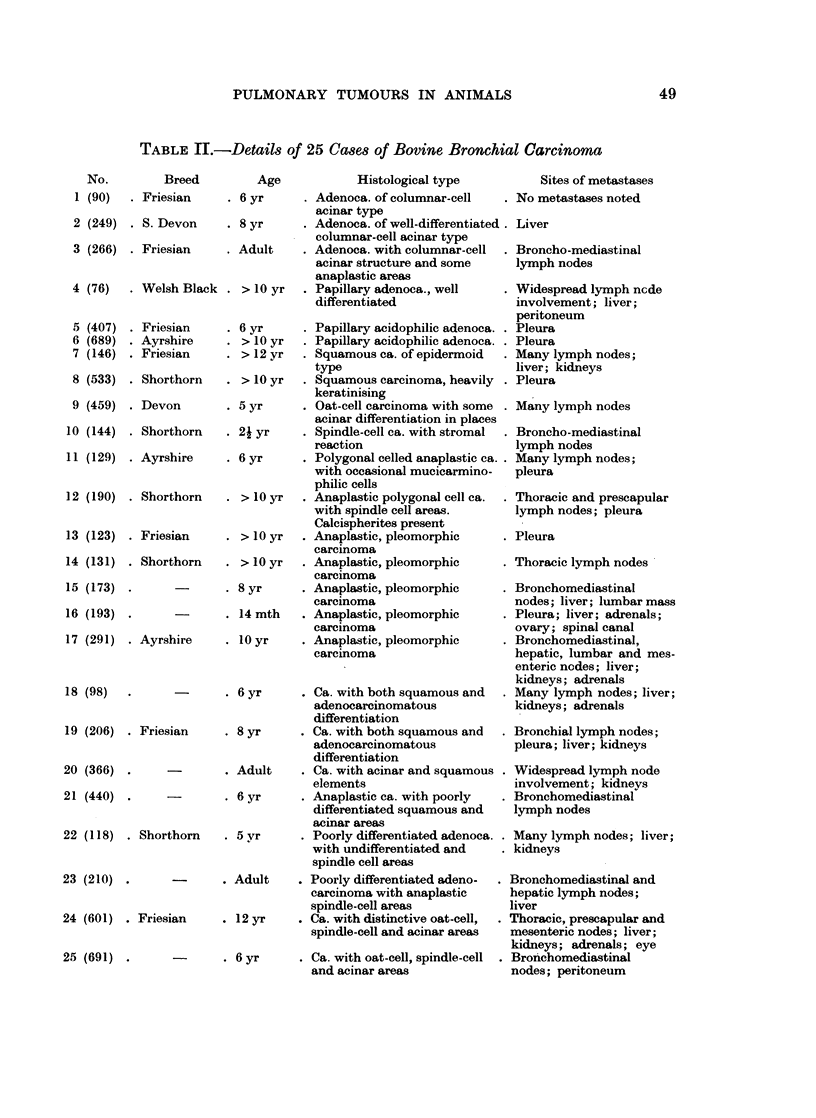

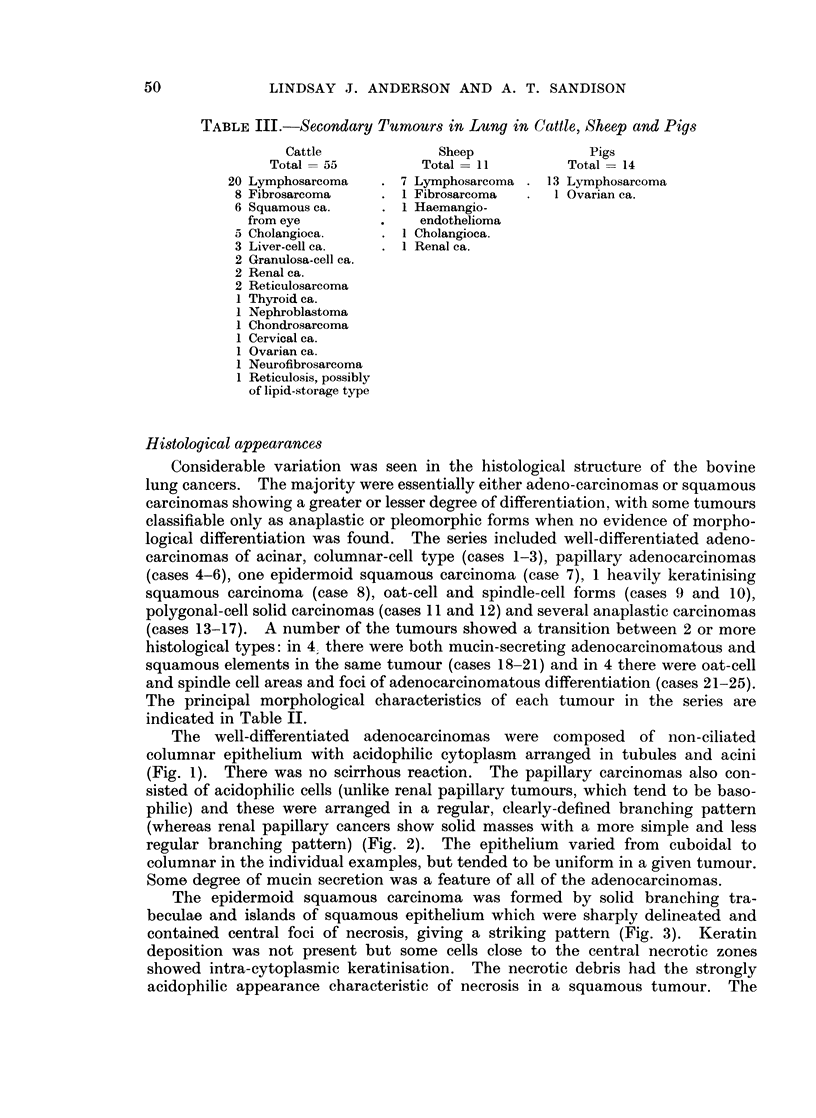

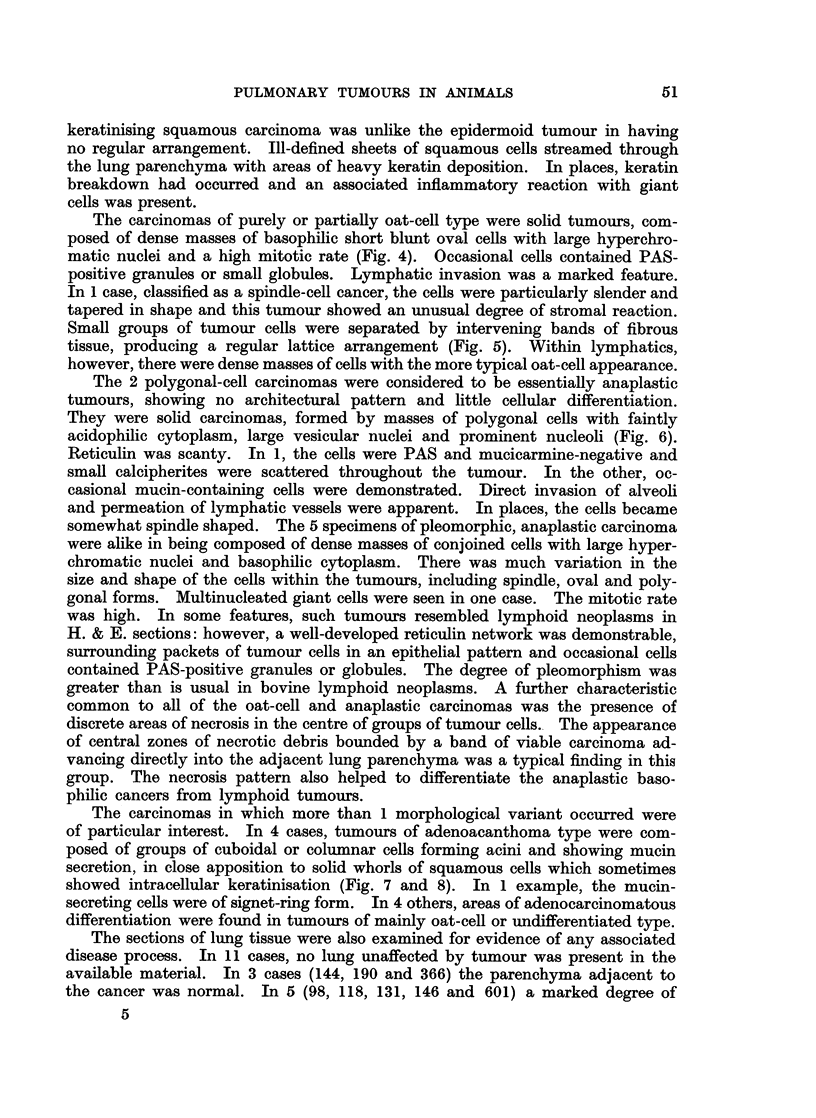

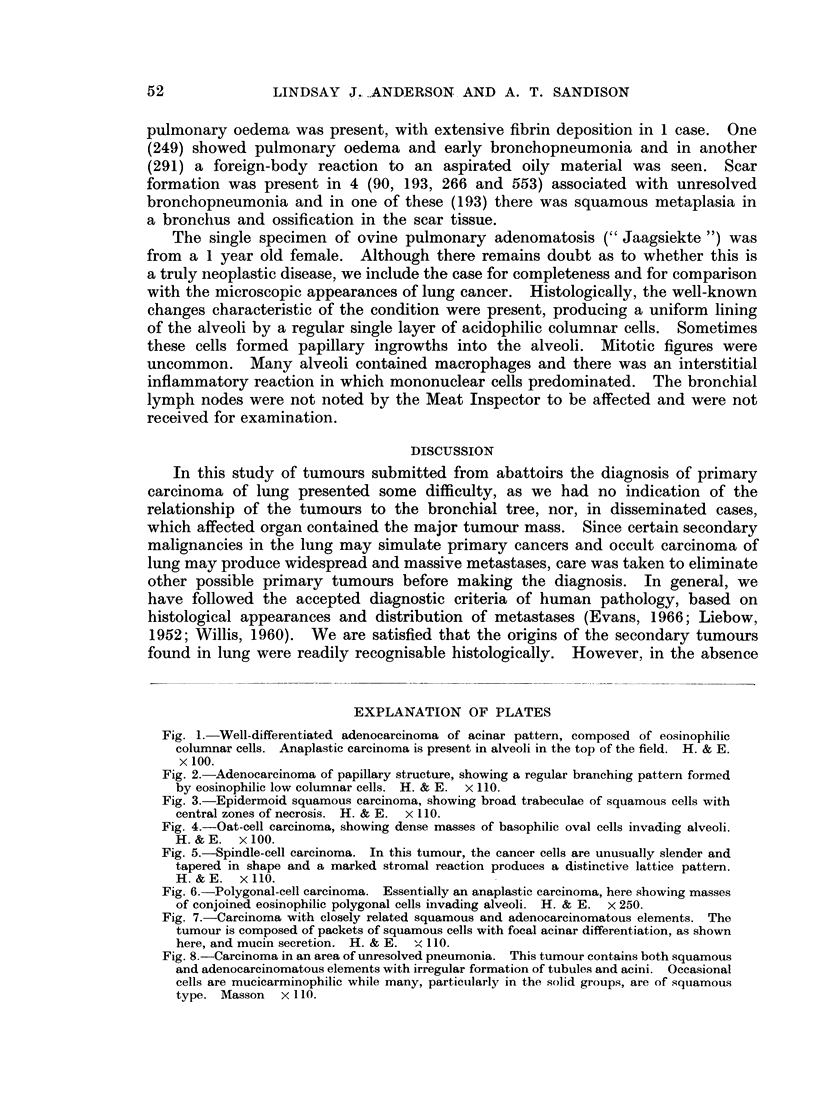

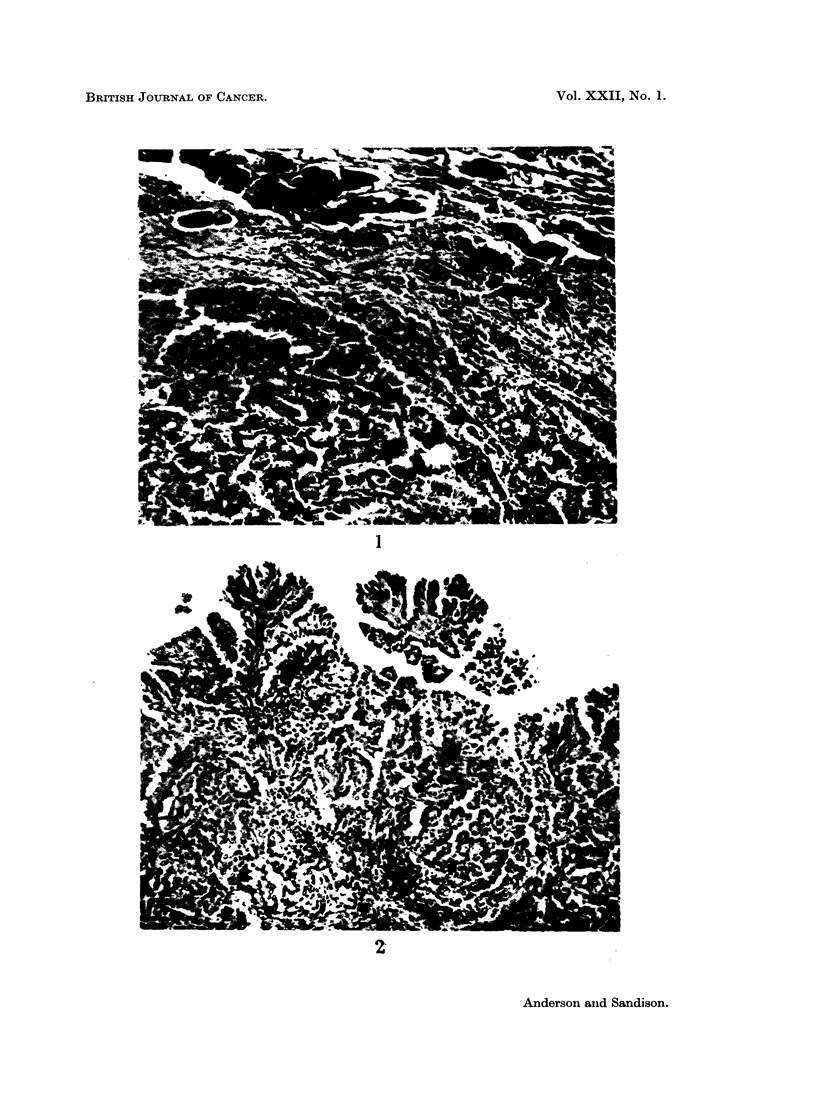

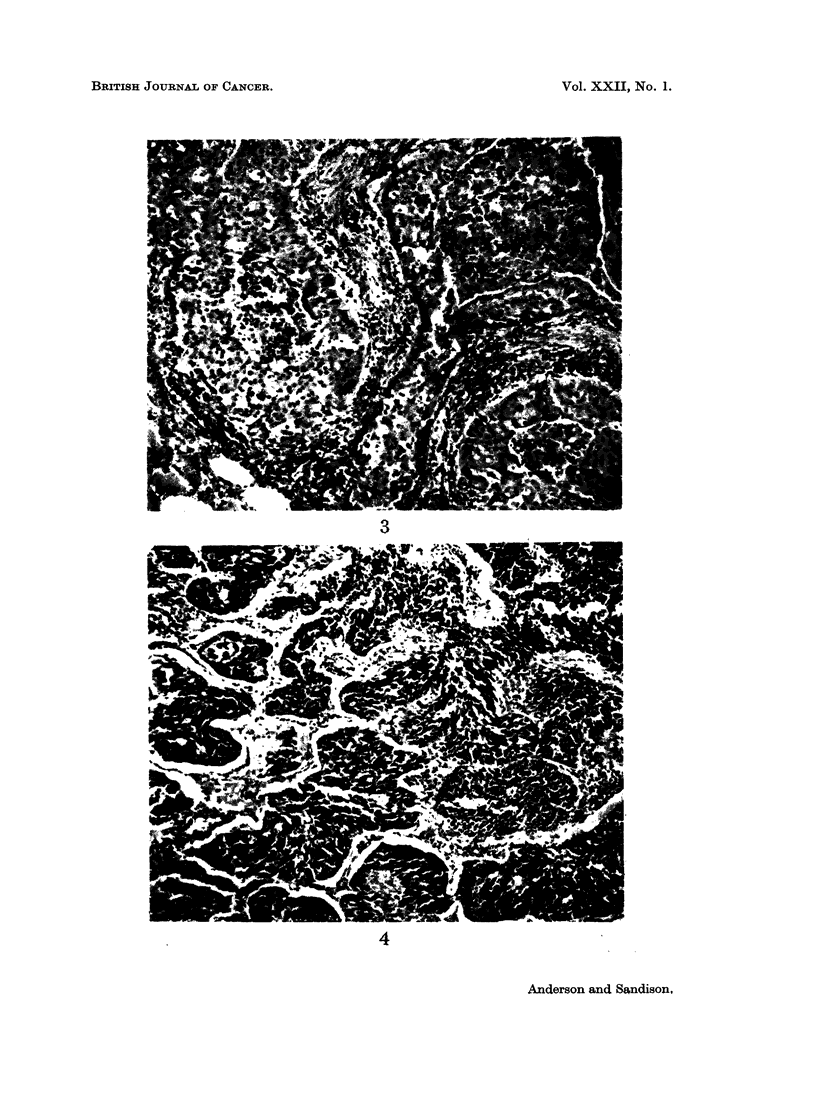

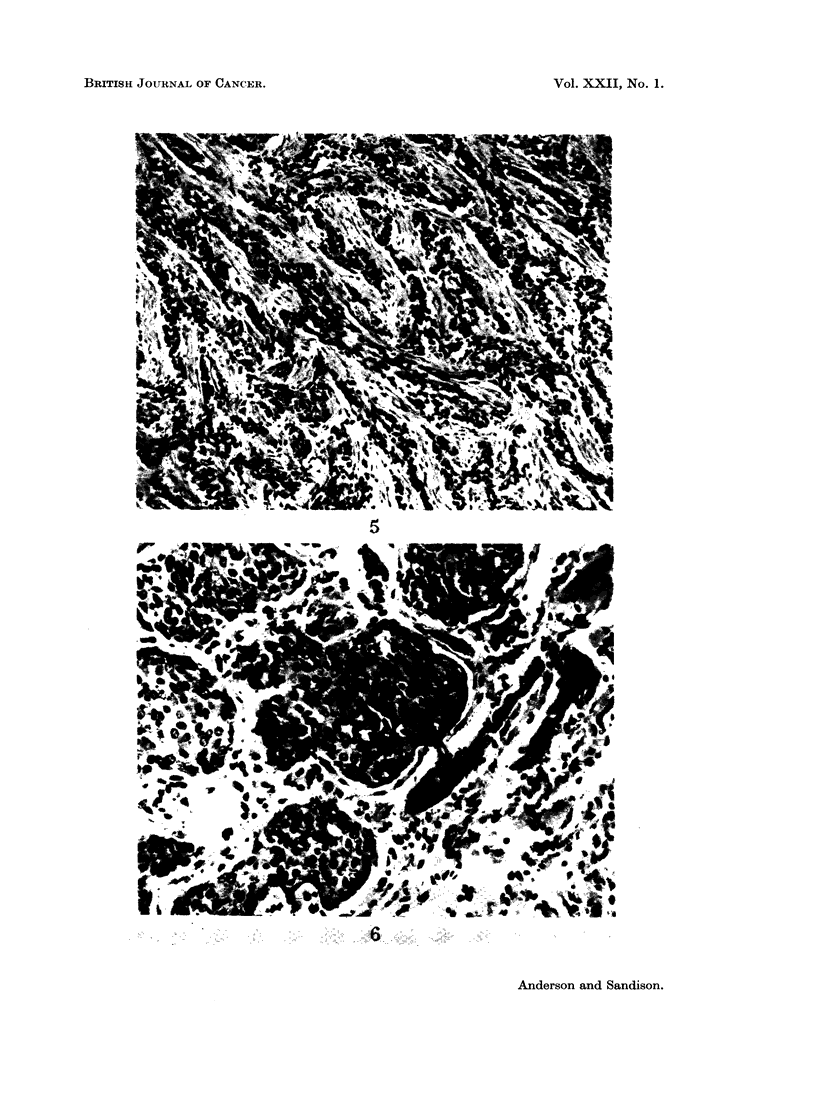

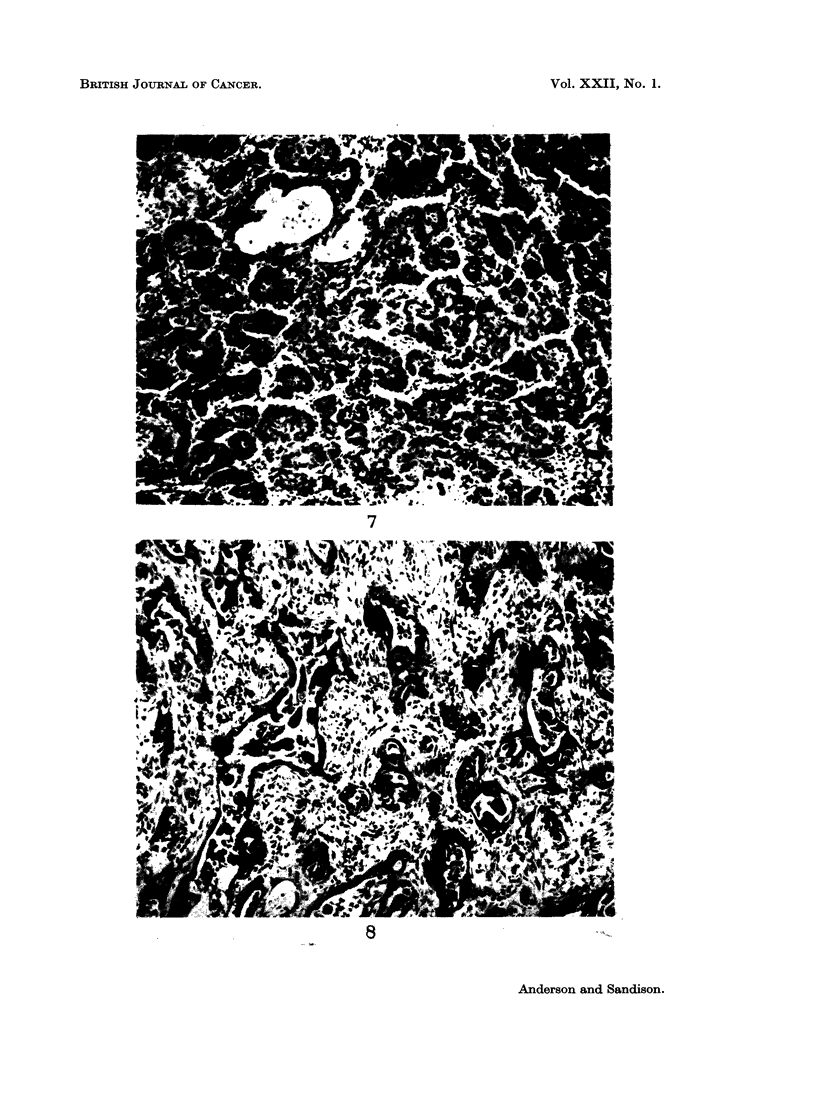

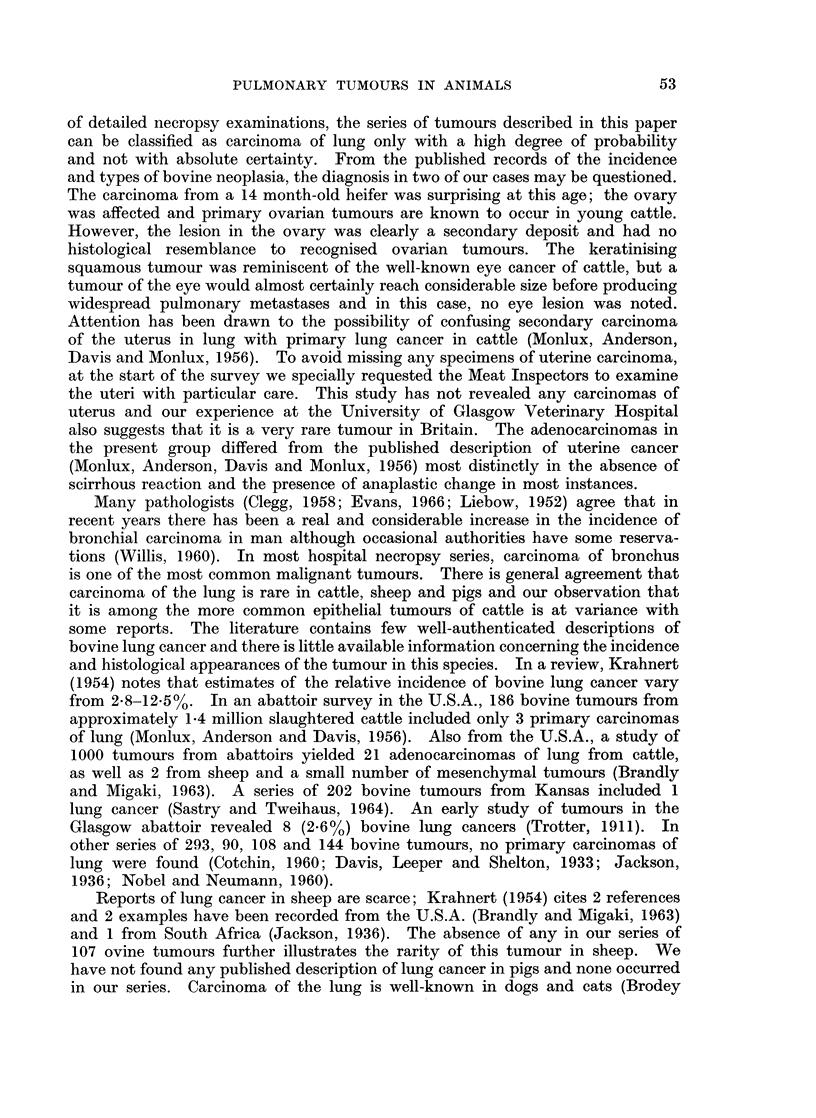

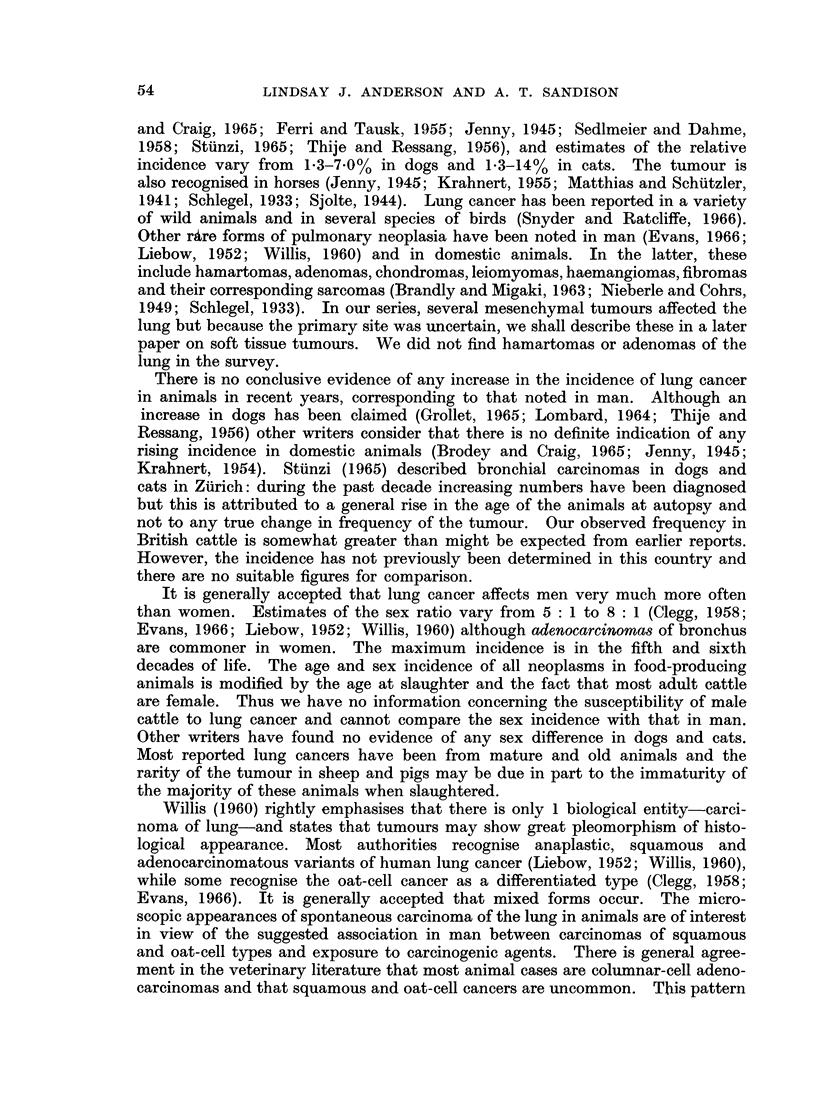

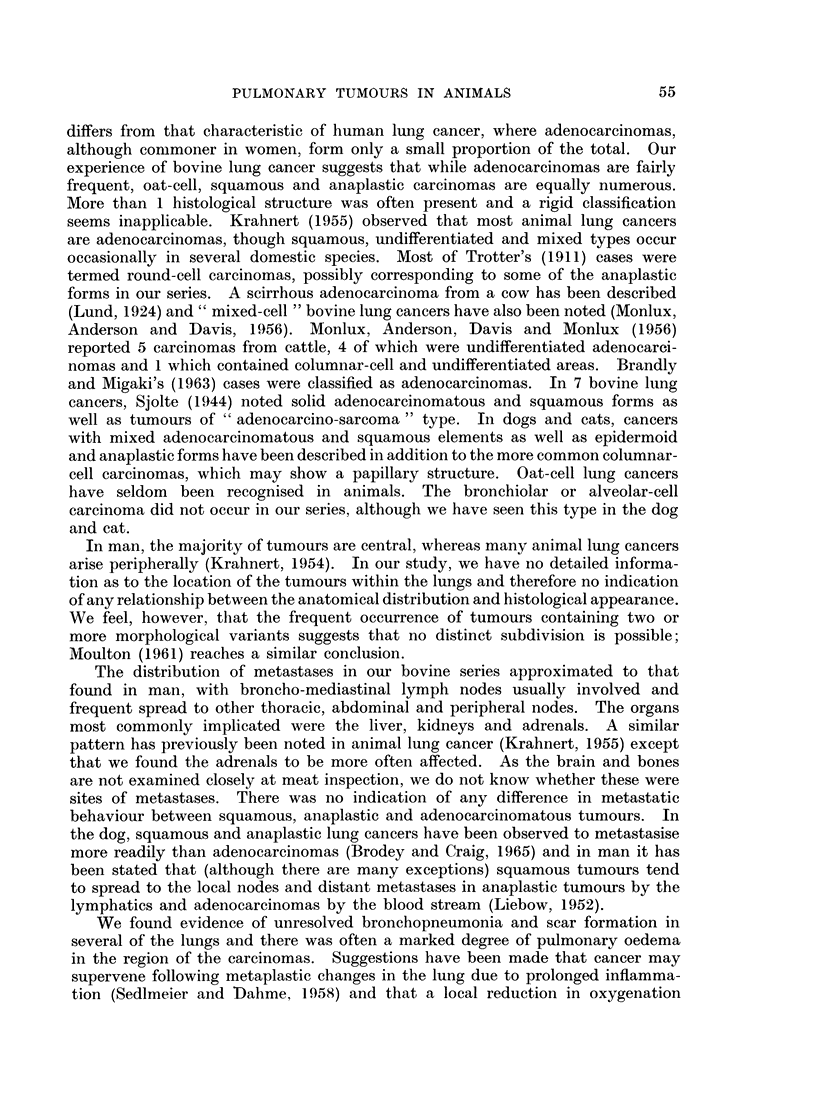

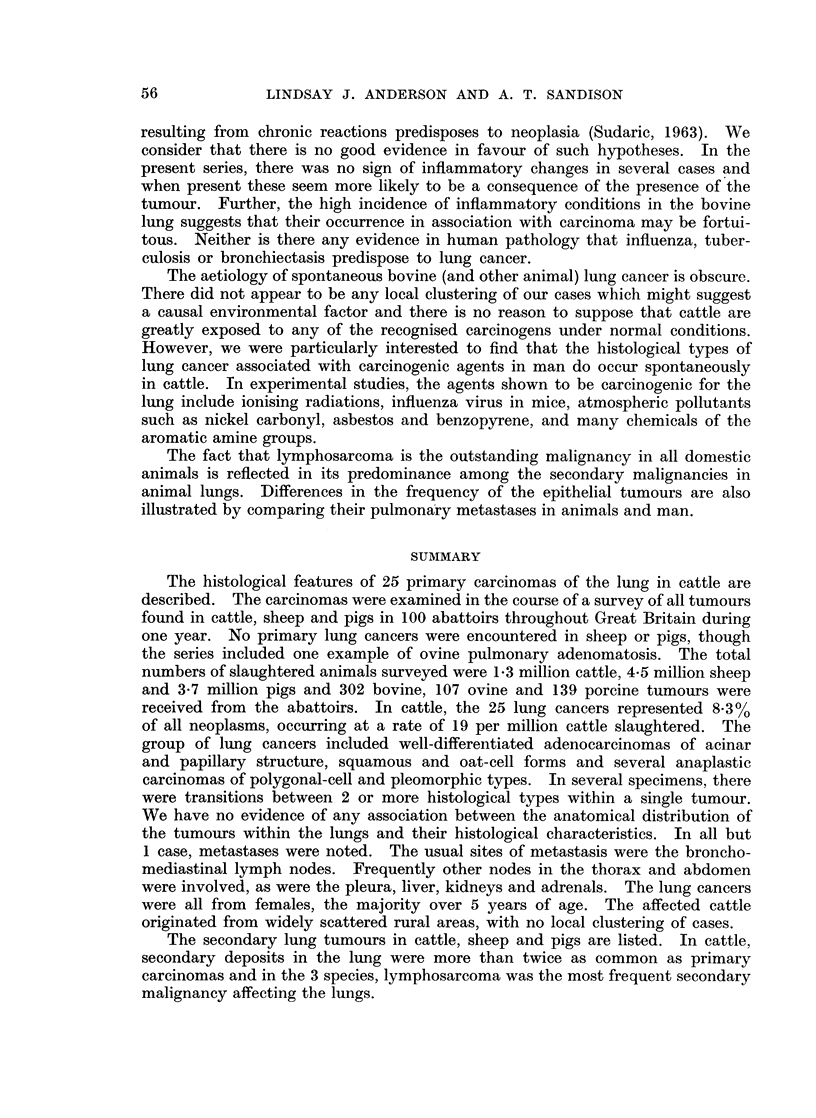

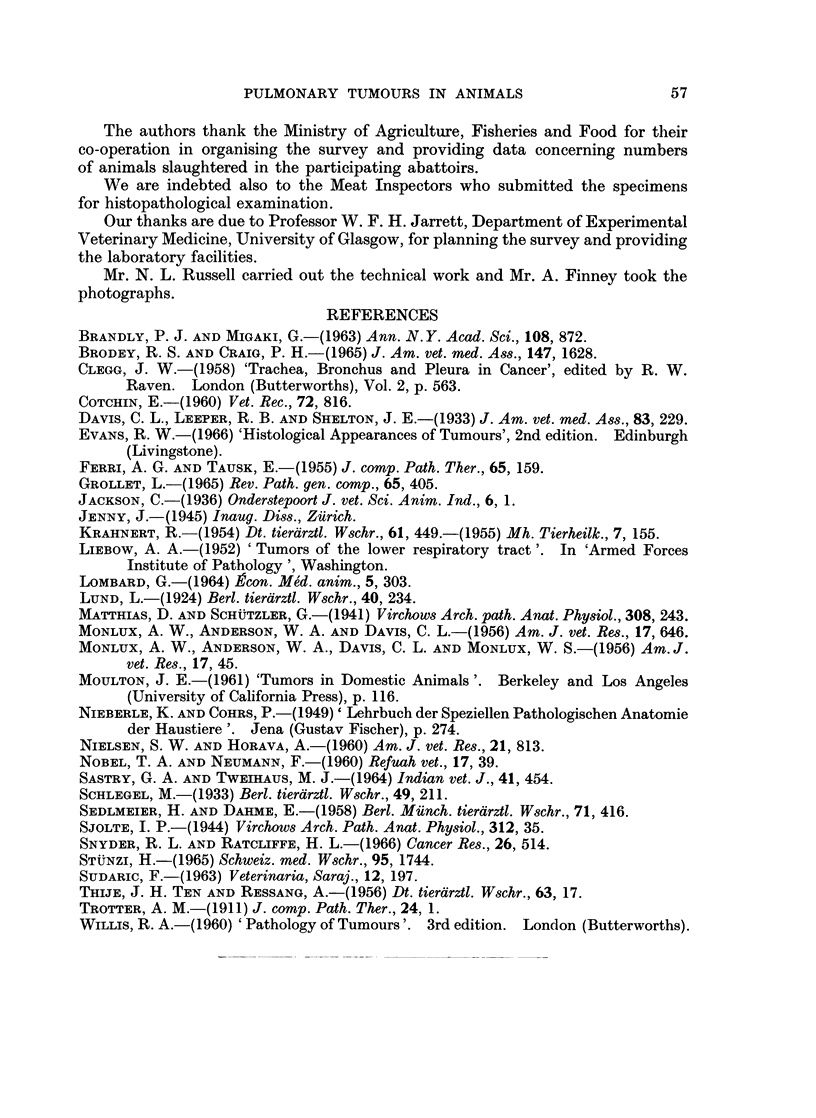


## References

[OCR_00905] ANDERSON W. A., DAVIS C. L., MONLUX A. W. (1956). A survey of tumors occurring in cattle, sheep, and swine.. Am J Vet Res.

[OCR_00878] Brodey R. S., Craig P. H. (1965). Primary pulmonary neoplasms in the dog: a review of 29 cases.. J Am Vet Med Assoc.

[OCR_00888] FERRI A. G., TAUSK E. (1955). Primary pulmonary carcinomas of the dog.. J Comp Pathol.

[OCR_00891] Grollet L. (1965). Progression du cancer du poumon chez l'homme et les animaux.. Rev Pathol Comp.

[OCR_00918] NIELSEN S. W., HORAVA A. (1960). Primary pulmonary tumors of the dog. A report of sixteen cases.. Am J Vet Res.

[OCR_00927] Snyder R. L., Ratcliffe H. L. (1966). Primary lung cancers in birds and mammals of the Philadelphia zoo.. Cancer Res.

